# The Rose (*Rosa hybrida*) NAC Transcription Factor 3 Gene, *RhNAC3*, Involved in ABA Signaling Pathway Both in Rose and *Arabidopsis*

**DOI:** 10.1371/journal.pone.0109415

**Published:** 2014-10-07

**Authors:** Guimei Jiang, Xinqiang Jiang, Peitao Lü, Jitao Liu, Junping Gao, Changqing Zhang

**Affiliations:** 1 Department of Ornamental Horticulture, College of Agriculture and Biotechnology, China Agricultural University, Beijing, PR China; 2 College of Landscape Architecture and Forestry, Qingdao Agricultural University, Qingdao, PR China; Key Laboratory of Horticultural Plant Biology (MOE), China

## Abstract

Plant transcription factors involved in stress responses are generally classified by their involvement in either the abscisic acid (ABA)-dependent or the ABA-independent regulatory pathways. A stress-associated NAC gene from rose (*Rosa hybrida*), *RhNAC3*, was previously found to increase dehydration tolerance in both rose and *Arabidopsis*. However, the regulatory mechanism involved in RhNAC3 action is still not fully understood. In this study, we isolated and analyzed the upstream regulatory sequence of *RhNAC3* and found many stress-related *cis*-elements to be present in the promoter, with five ABA-responsive element (ABRE) motifs being of particular interest. Characterization of *Arabidopsis thaliana* plants transformed with the putative *RhNAC3* promoter sequence fused to the β-glucuronidase (GUS) reporter gene revealed that *RhNAC3* is expressed at high basal levels in leaf guard cells and in vascular tissues. Moreover, the ABRE motifs in the *RhNAC3* promoter were observed to have a cumulative effect on the transcriptional activity of this gene both in the presence and absence of exogenous ABA. Overexpression of *RhNAC3* in *A. thaliana* resulted in ABA hypersensitivity during seed germination and promoted leaf closure after ABA or drought treatments. Additionally, the expression of 11 ABA-responsive genes was induced to a greater degree by dehydration in the transgenic plants overexpressing *RhNAC3* than control lines transformed with the vector alone. Further analysis revealed that all these genes contain NAC binding *cis*-elements in their promoter regions, and RhNAC3 was found to partially bind to these putative NAC recognition sites. We further found that of 219 *A. thaliana* genes previously shown by microarray analysis to be regulated by heterologous overexpression *RhNAC3,* 85 are responsive to ABA. In rose, the expression of genes downstream of the ABA-signaling pathways was also repressed in *RhNAC3*-silenced petals. Taken together, we propose that the rose RhNAC3 protein could mediate ABA signaling both in rose and in *A. thaliana*.

## Introduction

Drought, or dehydration, is one of the major limiting factors for plant growth, development, and productivity and plants have evolved a range of physiological, biochemical and molecular responses to promote drought stress tolerance [1]. One such response to drought stress is the production of the plant hormone abscisic acid (ABA), which mediates numerous downstream responses, including stomatal closure, thereby restricting water loss. Using genomic and transcriptomic analyses, the products of drought-inducible genes, including those regulated by ABA, have been classified into two groups: structural proteins and regulatory proteins [2], such as transcription factors (TFs).

Drought or dehydration-induced TFs have been isolated from many plant species and demonstrated to be involved in drought tolerance, such as *DREB2A*, *DREB2C*, *AREB1*, and *WRKY57* from *A. thaliana* [3], [4], *OsbZIP46* from *Oryza sativa* [5], *TaMYB30-B* from *Triticum aestivum* [6], *RhNAC2* from *Rosa hybrida* [7], and *ThbZIP1* from *Tamarix hispida* [8]. TFs involved in stress responses are typically classified as being involved either in ABA-dependent or the ABA-independent regulatory pathways [9] and structure and sequence analyses of the promoters of ABA-dependent TF genes have identified many stress-related *cis*-elements. These include the G-box (CACGTG, a MYC recognition site), the dehydration-responsive element/C-repeat (DRE/CRT) and the ABA-responsive element [10], [11]. Among these, the conserved ABA-responsive *cis*-element, PyACGTG/TGC, also named ABA-responsive element (ABRE), is a signature sequence for genes involved in the ABA signaling pathway [12], [13], and is important for promoter activity under osmotic stress conditions, such as those resulting from dehydration and high salinity [12], [14]. The functions of ABA-dependent TFs have also been investigated through overexpression in many plant species [11], which has been found to result in hypersensitivity to ABA during seed germination [5], [15], constitutive stomatal closure [16] and severely inhibited root growth [17]. Microarray analysis further revealed that constitutive overexpression of ABA-related TF genes, such as *ABO3* and *MYB96*, generally increases the expression of downstream ABA-responsive genes, and results in drought tolerance [18], [19].

Another class of ABA-related TFs are NAC (*N*AM, *A*TAF1 and 2, and *C*UC2) proteins, plant-specific transcriptional regulators that contain conserved N-terminal NAC domains and divergent C-terminal regions [20]. NAC TFs play important roles in regulating numerous aspects of growth and development, including cell division, and senescence, as well as responses to environmental stress stimuli [21]. Many are involved in ABA mediated signaling during their response to abiotic stresses, such as *A. thaliana* ANAC019 and ANAC055, soybean GmNAC011 and GmNAC020 and rice OsNAC5 [22], [23], [24], and their overexpression can result in enhanced ABA sensitivity at both the germination and post-germination developmental stages [25].

Different members of the NAC family have also been shown to be responsive to dehydration in rose petals [7]. Of these, *RhNAC2* has been found to promote petal cell expansion, in association with the regulation of cell wall-related genes [7], while *RhNAC3* regulates osmotic stress-related genes when exposed to drought stress [26]. However, nothing has been reported to date regarding the mechanism by which RhNAC3 participates in the ABA regulatory pathway. In this study, we characterized the upstream regulatory sequence of *RhNAC3* and found that ABREs in the *RhNAC3* promoter are needed for gene activity in both the presence and absence of exogenous ABA. Transgenic *A. thaliana* overexpressing *RhNAC3* showed enhanced ABA sensitivity during seed germination and during stomatal closure, and ABA-responsive genes were also up-regulated under dehydration conditions in both rose and the *A. thaliana* overexpressing *RhNAC* lines. These data indicate that ectopically expressed RhNAC3 enhances ABA sensitivity in *A. thaliana* and is involved in an ABA-dependent signaling pathway, at least some components of which are likely conserved between rose and *A. thaliana.*

## Materials and Methods

### Regulatory *cis*-element analysis

The upstream regulatory sequence of *RhNAC3* was isolated using PCR-based genome walking method [27]. (We state clearly that no specific permissions were required for these locations/activities and confirm that the field studies did not involve endangered or protected species). And the primers used are listed in Table S1. All amplified fragments were sub-cloned into the pGEM T-Easy Vector (Promega, Madison, WI, USA) and transformed into *Escherichia coli DH5a* cells after sequencing. The position of the translation start site was designated “0”. The *cis*-acting elements were analyzed and annotated using two software programs from the Plant *Cis*-acting Regulatory DNA Elements (PLACE) [28] (http://www.dna.affrc.go.jp/PLACE/) and Plant *Cis*-acting Regulatory Elements (PlantCARE) [29] (http://bioinformatics.psb.ugent.be/webtools/plantcare/html/) software programs. For NAC-binding site analysis of RhNAC3 upregulated genes in *A. thaliana*, a 1,000 bp regulatory sequence upstream of the genes was searched and analyzed by TAIR Loci Upstream Seq –1,000 bp of ‘Sequence Bulk Download and Analysis’ at www.arabidopsis.org.

### Construction of plant expression vectors and *Arabidopsis* transformation

The 977 bp upstream regulatory sequence of *RhNAC3* was amplified with 5′ ACC*AAGCTT*CATTCTACTTGTCCAAATCTGAACCTC 3′ and 5′ GCTCTAGACCGTATCAGAGAGATGAAACAGGAA 3′ (Table S1) and the product digested with *Hind* III and *Xba*I, and inserted into the pBI121 binary vector. The resulting Pro*_RhNAC3_*:GUS plasmid was introduced into the *Agrobacterium tumefaciens* strain *GV3101* and transformed into *A. thaliana* (Columbia) by the floral dip method [30]. Ten independent lines of kanamycin-resistant transgenic plants were obtained. The homozygous T3 generation seeds of the transgenic lines were used for subsequent experiments.

### Histochemical staining and quantitative GUS activity assay

Histochemical staining for GUS activity was performed as described by Li *et al*. (2009) [31]. Plant samples exposed to different treatments were immersed in GUS staining buffer (0.5 mM 5-bromo-4-chloro-3-indoly-β-D-GlcA, 0.5 M NaH_2_PO_4_, pH 7.0, 1 mM EDTA, 0.5 mM potassium ferricyanide and 0.5 mM potassium ferrocyanide). After staining at 37°C for 3–10 h, the samples were immersed in 95% (v/v) ethanol at 37°C to remove chlorophyll. For histochemical analysis of the Pro*_RhNAC3_*:GUS transgenic *A. thaliana* plants in response to ABA treatment, 9-day-old seedlings were grown on MS medium supplemented with 100 µM ABA for 4 days, before being sampled for histochemical GUS staining. The GUS staining patterns were examined under a microscope (BX51; Olympus) and analyzed using Photoshop CS6 software (Adobe, McLean, VA). Quantitative assays of GUS activity were performed as described by Jefferson *et al*. (1987) [32]. All experiments were performed three times to give three independent biological replicates.

### Construction of the truncated *RhNAC3* promoter-GUS fusion and transient expression assays

Three truncated *RhNAC3* promoter fragments, N0 (−1447 to −160 bp), N1 (−707 to −160 bp) and N2 (−377 to −160 bp) were amplified from rose genomic DNA and cloned into a modified pUC19 plasmid containing the GUS reporter gene, as described by Dai *et al.* (2012) [7]. To mutate the ABRE cis-element, we replaced the ACGT of the ABRE core sequence with TTTA using overlap PCR methods [33]. Mutation fragments of N0 (mN0, five ABREs mutated) and N1 (mN1, three ABREs mutated) were amplified, and cloned into the modified pUC19 as described for N0 and N1. *A. thaliana* mesophyll protoplasts were transformed with the resulting vectors: N0, mN0, N1, mN1 and N2, and an empty (normal) vector control (NC). For the ABA treatment experiments, *Arabidopsis* mesophyll protoplasts harboring the different constructs (N0, N1, N2 and NC) were exposed to 10 µM ABA (Sigma, St. Louis, MO). GUS activity was measured in protoplast extracts after 24 h of incubation with ABA. Isolation of *A. thaliana* mesophyll protoplasts, transformation of protoplasts and GUS activity assays were carried out as previously described [32], [34]. The primers are listed in Table S1, and the experiments were performed in triplicate.

### Seed germination assay and root growth measurements

The *RhNAC3*-overexpressing plants (overexpressor OE#3, OE#6 and OE#12) had previously been generated [26], and wild type (WT) and vector (VC) plants were used as controls. Approximately 50 seeds were plated onto solid MS medium supplemented with either 0, 0.2, 0.4 or 0.8 µM ABA. After vernalization at 4°C for 3 days, the seeds were moved to a temperature controlled room at 23±1°C under long-day conditions (16 h light/8 h dark cycle), with a light intensity of 80–100 µmol/m^2^/s and 40–60% relative humility. The rates of radicle emergence and cotyledon greening were measured after 7 days. All experiments were performed in triplicate. To measure seedling root growth, 5-day old seedlings of OE#3, OE#6 and OE#12 were transferred to plates of MS medium containing 5, 10, or 30 µM ABA respectively, and grown vertically. After growth for 10 d, primary root length and lateral root number was measured and analyzed using the Image J software (http://rsbweb.nih.gov/ij/). The WT and VC plants were used as controls.

### Stomatal aperture measurements

For ABA-induced stomatal closure, mature leaves from light-grown 3-week-old control and *RhNAC3* transgenic plants were detached and incubated in stomatal opening solution (10 mM KCl, 100 µM CaCl_2_ and 10 mM MES, pH 6.1) for 2 h at 22°C [16], before being transferred to fresh stomatal opening solution containing 0 µM or 10 µM ABA. Stomata on abaxial surfaces were photographed through a light microscope (BX51; Olympus), and the stomatal aperture (the ratio of width to length) was measured (n = 20). For drought-induced stomatal closure, 3-week-old seedlings of WT, VC and *RhNAC3*-overexpressing lines were grown for 10 d without water. Plants grown under normal conditions were used as control. Leaves in the same position on the plant were sampled, and the stomata on the leaf abaxial surfaces were immediately photographed. Stomatal aperture was measured (n = 20) and all experiments were repeated three times.

### Quantitative reverse transcription PCR analysis

The detached leaves of 3-week-old vector (VC) and *RhNAC3* overexpressor (OE#3, 6 and 12) *A. thaliana* plants were dehydrated for 3 h at 23–25°C, 40–50% relative humidity, and 100 µmol m^−2^s^−1^ light intensity, then sampled for quantitative reverse transcription polymerase chain reaction (qRT-PCR) analysis. Total RNAs were isolated from the leaf samples using the Trizol agent (Invitrogen, Carlsbad, CA). DNase-treated RNA (1 µg) was used for first-strand cDNA synthesis (Invitrogen, Carlsbad, CA) and the cDNA (2 µL) was used as the template in a 20 µL qRT-PCR using a qPCR Kit (Kapa Biosystems, Woburn, MA). The *A. thaliana Actin2* gene (GenBank accession no. NM_112764) was used as an internal control. The 11 selected genes and gene specific primers used for the qRT-PCR analysis are listed in Table S1. Each qRT-PCR evaluation was performed with three biological replicates.

For qRT-PCR analysis of downstream genes of *RhNAC3* action in rose, nine putative ABA signaling and downstream rose genes from the ABA-signaling pathways were selected from our rose transcriptome databases [7] (Table S2). The rose cDNAs from Tobacco Rattle Virus (TRV) and *RhNAC3*-silenced petals were obtained in our previous study [26]. *RhUbi1* (accession no. JK622648) was used as the internal control. The gene specific primers for qRT-PCR are listed in Table S1. Each qRT-PCR analysis was performed with three biological replicates.

### Electrophoretic mobility-shift assay

The electrophoretic mobility-shift assay (EMSA) was performed according as previously described [26] with minor modifications. To construct the GST-RhNAC3 fusion protein, the N-terminal of RhNAC3 (RhNAC3^N1–162^) was amplified by PCR (primers are listed in Table S1) and the PCR product was ligated into the pGEX-2T vector (Pharmacia LKB Biotechnology, Piscataway, NJ) via the *Bam*HI and *Sac*I sites and the recombinant vector was expressed in *Escherichia coli* BL21 cells. The fusion protein was induced by 0.2 mM isopropyl β-D-1-thiogalactopyranoside (IPTG), and the cells *E. coli* incubated at 28°C for a further 6 h. The recombinant protein was purified by GST-agarose affinity chromatography (GE Healthcare, http://www.gehealthcare.com/). Biotin-labeled DNA fragments used in the EMSA contain one or two putative NAC binding sequences [21]. The probes were incubated with the fusion protein at room temperature for 25 min in binding buffer (10× concentration: 100 mM Tris, 500 mM KCl, 10 mM dithiothreitol; pH 7.5). Each 20 µL binding reaction contained 0.2 pmol biotin probe and 2 µg fusion protein, and 1 µg Poly (dI•dC) was added to the reaction to minimize nonspecific interactions. The reaction products were analyzed using 5% native polyacrylamide gel electrophoresis and 0.5× Tris-borate/EDTA buffer. After electrophoresis, the DNA fragments on the gel were transferred to a nitrocellulose membrane using 0.5× Tris-borate/EDTA at 380 mA (∼100 V) for 30 min at 4°C. After UV cross-linking, the membrane was transferred to conjugate/blocking buffer by mixing 16.75 µL stabilized streptavidin-horseradish peroxidase conjugate with 5 mL blocking buffer. After washing, biotin-labeled DNA was detected by chemiluminescence according to the manufacturer’s protocol (Pierce, http://www.piercenet.com/).

## Results

### Structure and sequence analysis of the *RhNAC3* promoter

**Figure 1:**
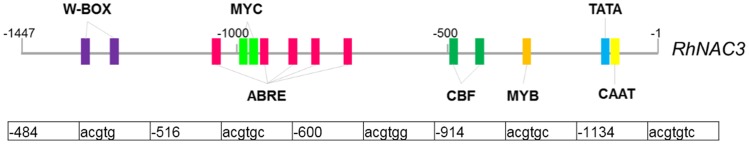
Schematic representation of the *RhNAC3* promoter. The major stress-related *cis*-acting elements in the 1447 bp promoter of *RhNAC3* are shown. The position and putative sequences of ABRE elements are listed.

To elucidate the regulation of *RhNAC3* transcription, a 1,447 bp fragment corresponding to the sequence immediately upstream of its translational start site (TSS) (GenBank accession number: KJ000025) was isolated by PCR-based genome walking. Subsequent sequence analysis of this region revealed a number of putative *cis*-elements, including elements associated with ABA, cold, pathogen and wounding responses (Figure 1, Figure S1 and Table S2). The TATA box (TTATTT) was found −104 bp upstream of the TSS and a CAAT-box sequence (CAAT) was found 2 bp downstream of the TATA-box sequence. Five ABRE-related sequence motifs (ACGTG), located at positions −1140 to −1136, −918 to −914, −605 to −601, −521 to −517 and −488 to −484, were identified, which we hypothesized might be involved in an ABA response. Other potential regulatory elements that were found include two CBF sequences (RYCGAC) at positions −493 to −488 and −404 to −399, and a Myb-type TF recognition sequence (GGATA) at position −291 to −287. There are also two Myc-type TF recognition sequences (CACATG) at positions −985 to −980 and −951 to −946, and two W-box sequences (TGACT) at positions −1300 to −1296 and −1231 to −1227, relative to the TSS. Collectively, the presence of these *cis*-elements suggests that *RhNAC3* may play a role in responses to a variety of stresses, such as cold, pathogen challenge or wounding, particularly via the ABA-dependent pathway.

### Activity of the *RhNAC3* promoter in Pro*_RhNAC3_*:*GUS* transgenic *A. thaliana* lines

**Figure 2:**
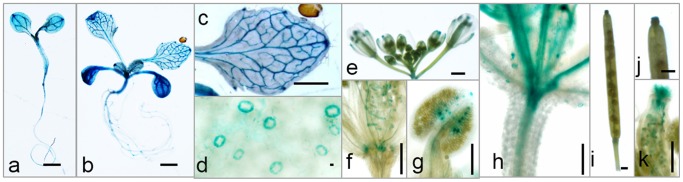
Histochemical analysis of *RhNAC3* expression in *A. thaliana*. The *GUS* gene driven by the *RhNAC3* promoter was expressed in 5-day-old *A. thaliana* seedlings (**a**), 14 day-old seedlings (**b**), young leaves (**c**), stomata of young leaves (**d**), flowers (**e**), petals (**f**), stigma (**g**), apical stems of inflorescences (**h**), mature siliques (**i** and **j**; the latter is magnified); and immature seeds (**k**), Scale bars = 1 mm.

We next examined the spatial expression pattern of the *RhNAC3* gene in 10 independent *A. thaliana* lines (Pro*_RhNAC3_*:*GUS*) that had been transformed with a construct containing the *RhNAC3* promoter fused to the *GUS* reporter gene. The T3 generation homozygotes of six lines were selected to analyze by GUS staining. Histochemical GUS staining revealed that Pro*_RhNAC3_*:*GUS* was expressed almost throughout the entire plant during the seedling stage of development (Figure 2a and b), and particularly strong staining was observed in the vascular system and leaf stomatal guard cells (Figure 2c and d). In addition, GUS staining was detected in the flower petals (Figure 2e and f), stigma (Figure 2g) and apical stem of the inflorescence (Figure 2h), while in mature siliques, staining was primarily localized to the stigma and immature seeds (Figure 2i, j and k). We conclude that the *RhNAC3* gene was expressed ubiquitously in a number of different plant tissues, with higher basal expression levels in leaf guard cells and areas of the vascular system.

### ABREs are important for *RhNAC3* promoter activity

**Figure 3:**
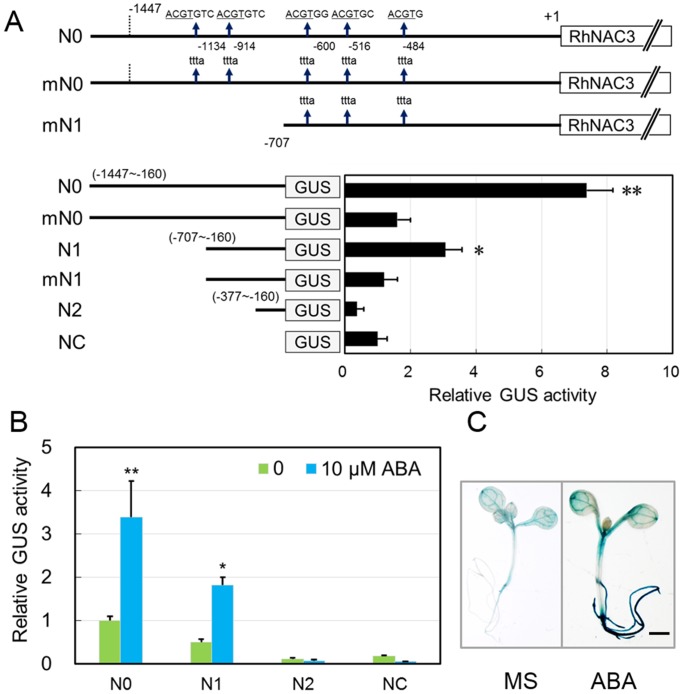
Deletion analysis and ABA dose dependent response of *RhNAC3* promoter activity. **A,** Assays of GUS activity in *A. thaliana* protoplasts containing RhNAC3 promoter deletion and ABRE mutation constructs. The numbers on top represent the positions of the ABRE *cis*-elements and mutations of ABREs in the *RhNAC3* promoter region. Relative GUS activity in transient expression experiments using five different constructs (N0, mN0, N1, mN1 and N2) and the vector control (NC) is shown at the bottom. GUS activity was determined after 24 h of incubation. Error bars represent standard error (*n* = 3). **: *P*<0.01, *: *P*<0.05, *t* test. **B,** Effects of exogenous ABA on GUS activity in *A. thaliana* protoplasts containing *RhNAC3* promoter deletion constructs. The truncated *RhNAC3* promoter constructs (N0, N1 and N2) and vector control (NC) were transformed into *A. thaliana* protoplasts, which were then exposed to 0 and 10 µM exogenous ABA. GUS activity in protoplast extracts was measured after 24 h of incubation with ABA. Error bars represent standard error (*n* = 5). **C,** Histochemical analysis of *RhNAC3* promoter::GUS expression in response to ABA. 9-day-old transgenic seedlings were grown on MS medium only or MS medium plus ABA (transferred to MS medium plus 100 µM ABA for 4 days) before being subjected to histochemical GUS staining. Scale bar = 1 mm.

To assess the potential role of ABREs in the transcriptional activity of the *RhNAC3* promoter, we made three truncated promoter fragments containing five (N0), three (N1) or no (N2) ABREs, and two fragments, mN0 and mN1 with replacement of ACGT by TTTA in ABRE core sequence. These fragments were fused to the *GUS* reporter gene in the plant transient expression vector pUC19 (Figure 3A, top). The resulting constructs, as well as the vector control (NC), were transformed into *A. thaliana* protoplasts and relative GUS activity was measured. We observed that extracts from the protoplasts transformed with constructs containing a higher number of ABRE copies had higher GUS activity than control transformant extracts. Specifically, the N0 and N1 construct extracts had 7.2-fold and 3.1-fold greater GUS activity, respectively, than the NC extract, while constructs with ABRE mutations (mN0 and mN1) conferred only slight GUS activity and minimal activity was detected in the N2 construct extract (Figure 3A, bottom). We also investigated the transcriptional activity of three truncated *RhNAC3* promoter fragments in *A. thaliana* protoplasts exposed to exogenous ABA. Transcription of the *RhNAC3* promoter (N0 and N1) was found to be induced with a higher GUS activity for the N0 fragment (five ABREs) than for N1 (three ABREs), while no difference was seen for N2 (no ABRE) when ABA was added (Figure 3B). The potential role of the ABREs in the *RhNAC3* promoter in ABA induced transcription was also evaluated using the Pro*_RhNAC3_*:*GUS* transgenic *A. thaliana* lines. Seedlings of thee lines had stronger GUS staining after treatment with ABA than those without ABA treatment (Figure 3C). Taken together these data indicate that ABREs in the *RhNAC3* promoter have a cumulative effect on the transcription activity of *RhNAC3* both in the presence and absence of ABA, and that ABA significantly induces *RhNAC3* transcription.

### *A. thaliana* plants overexpressing *RhNAC3* show hypersensitivity to ABA during germination

**Figure 4:**
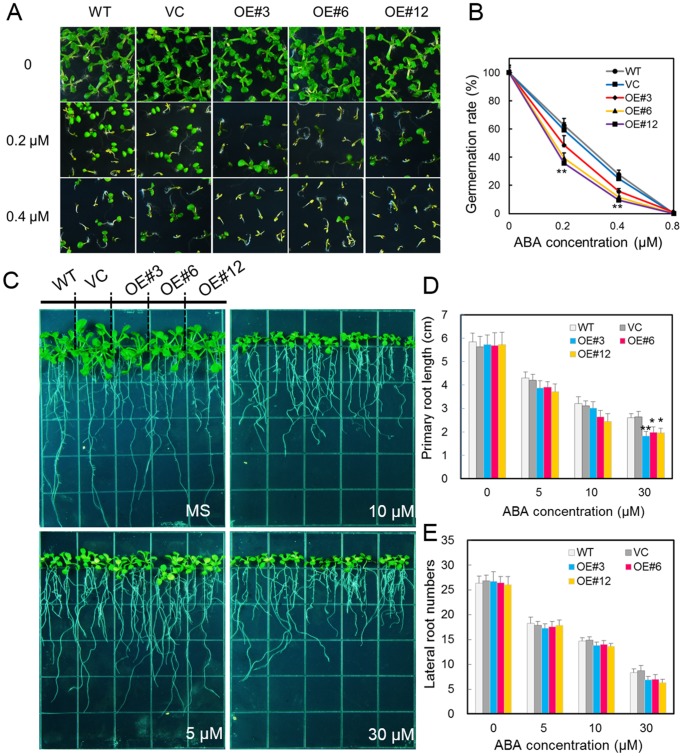
Effect of ABA concentration on seed germination and root growth in WT and *RhNAC3* overexpressing *A. thaliana* plants. **A,** Seed germination phenotypes. The homozygous T3 seeds of *RhNAC3*-overexpressing lines (OE#3, OE#6 and OE#12), wild type (WT) and vector plants were plated on MS supplemented with 0, 0.2 or 0.4 µM ABA. Images were obtained 7 days after planting. **B,** Seed germination rates. The germination rates were measured 7 days after planting. Error bars represent standard error (*n* = 3). **: *P*<0.01, *: *P*<0.05, *t* test. **C,** Root growth phenotypes. Five-day-old seedlings of WT, vector only and three *RhNAC3*-overexpressing *A. thaliana* lines (OE#3, #6 and #12) were transferred to MS plates supplemented with 0, 5, 10 and 30 µM ABA. Root phenotypes were visualized 10 days after planting. **D,** Primary root length analysis. **: P<0.01, *: P<0.05, *t* test. **E,** Lateral root number analysis. Both primary root length and lateral root number were measured after 10 days of growth. Three independent experiments were performed using 15 plants in each experiment in D and E. Error bars represent standard error (*n* = 3).

Our previous study showed that three representative *RhNAC3*-overexpressing *A. thaliana* lines (OE#3, OE#6 and OE#12) had enhanced drought tolerance, with a higher water-retaining ability [26]. To understand the roles of *RhNAC3* in the ABA signaling pathway, we investigated the seed germination rates of *RhNAC3* overexpressing lines following ABA treatment. More than 98% of the seeds sown on control MS medium germinated well, while the germination rate of both the control and transgenic seeds decreased when grown for 7 days on MS medium plus ABA. In the presence of 0.2 µM ABA, the germination rates of WT and Vector (VC) seeds were 63% and 59%, while the rates for OE#3, OE#6 and OE#12 were 49%, 40% and 36%, respectively. A higher concentration of ABA (0.4 µM) resulted in lower germination rates for both the control and *RhNAC3*-overexpressing transgenic plants, and the latter showed a greater decrease (Figure 4A and 4B). We also compared the effects of ABA on the root architectures of the *RhNAC3*-overexpressing and control plants. Primary root growth of *RhNAC3*-overexpressors was inhibited more by 30 µM ABA treatment than that of the control plants, while no significant differences in the number of lateral roots was observed (Figure 4C–E). We conclude from these results that *RhNAC3* overexpression in *A. thaliana* results in ABA hypersensitivity at the seed germination stage.

### RhNAC3 participated positively in ABA- and drought-induced stomatal closure

**Figure 5:**
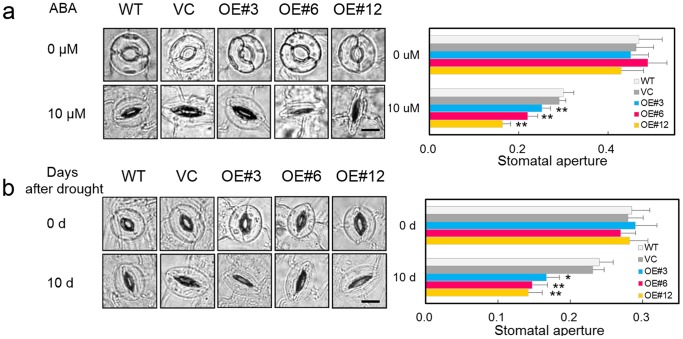
Stomatal aperture of the *RhNAC3* overexpressing *A. thaliana* plants in response to ABA and drought treatments. **A,** Stomatal aperture in response to ABA. Mature leaves from three-week old wild type (WT), vector control and independent *RhNAC3*-overexpressing plants (OE#3, OE#6 and OE#12) were treated with a stomatal opening solution for 2 h (0 µM) and incubated with 10 µM ABA for 2 h (10 µM). Stomata on the abaxial surfaces were imaged by light microscopy. Stomatal aperture (the ratio of width to length) was quantified using at least 20 guard cells from each sample. *Bar* 10 µm. **B,** Stomatal aperture of *RhNAC3* overexpressing lines in response to drought stress. Three-week old seedlings of WT, vector control and independent *RhNAC3*-overexpressing plants (OE#3, OE#6 and OE#12) were subjected to 10 days without water. Plants grown under normal well watered conditions were used as a control. The leaves were harvested and the stomata on the leaf abaxial surfaces were immediately photographed. Stomatal apertures were then quantified (n = 20). *Bar* 10 µm. **: *P*<0.01, *: *P*<0.05, *t* test.

Since the leaves of *RhNAC3*-overexpressing *A. thaliana* plants have greater water-retaining capacity than those of WT plants [26], we examined ABA-dependent stomatal movement phenotypes. Expanded leaves of 3-week-old plants (12 h day/12 h night) were submerged in stomatal opening solution, treated with 10 µM ABA for 2 h, and then the stomatal apertures of the guard cells were measured in the focal planes of the outer edge in epidermal strips. None of the plants exhibited altered stomatal movement in the absence of ABA, and most of the guard cells examined were fully opened. No obvious difference in stomatal aperture (the ratio of width to length) were observed between the controls (WT and VC) and *RhNAC3* overexpressors (OE#3, OE#6 and OE#12). However, in the presence of 10 µM ABA, stomatal closure in the leaves of *RhNAC3* overexpressing transgenic plants was substantially enhanced compared with that of control plant leaves. The stomatal aperture ratios in the OE#3, OE#6, and OE#12 lines were approximately 0.16, 0.22 and 0.25, respectively, compared with 0.30 and 0.29 for the WT and VC plants (Figure 5A).

We also investigated stomatal movement in the leaves of *RhNAC3-*overexpressing *A. thaliana* plants grown under drought conditions. Water was withheld for 10 days from three-week-old plants that had previously been grown under normal conditions, after which the stomatal apertures of the expanded leaves were measured. Under normal growth conditions (0 days), both the controls (WT and VC) and overexpressor lines (OE#3, OE#6 and OE#12) showed no obvious difference, with stomatal apertures of 0.29, 0.28, 0.29, 0.27 and 0.28, respectively. However, after 10 days of exposure to drought conditions, the stomatal aperture ratios had decreased to 0.17, 0.15 and 0.12 in OE#3, OE#6 and OE#12 lines, respectively, but only 0.24 in WT and 0.23 in VC plants (Figure 5B). These results indicate that RhNAC3 is involved in stress responses in an ABA-dependent manner.

### RhNAC3 activates ABA-responsive gene expression in rose and *Arabidopsis*

**Figure 6:**
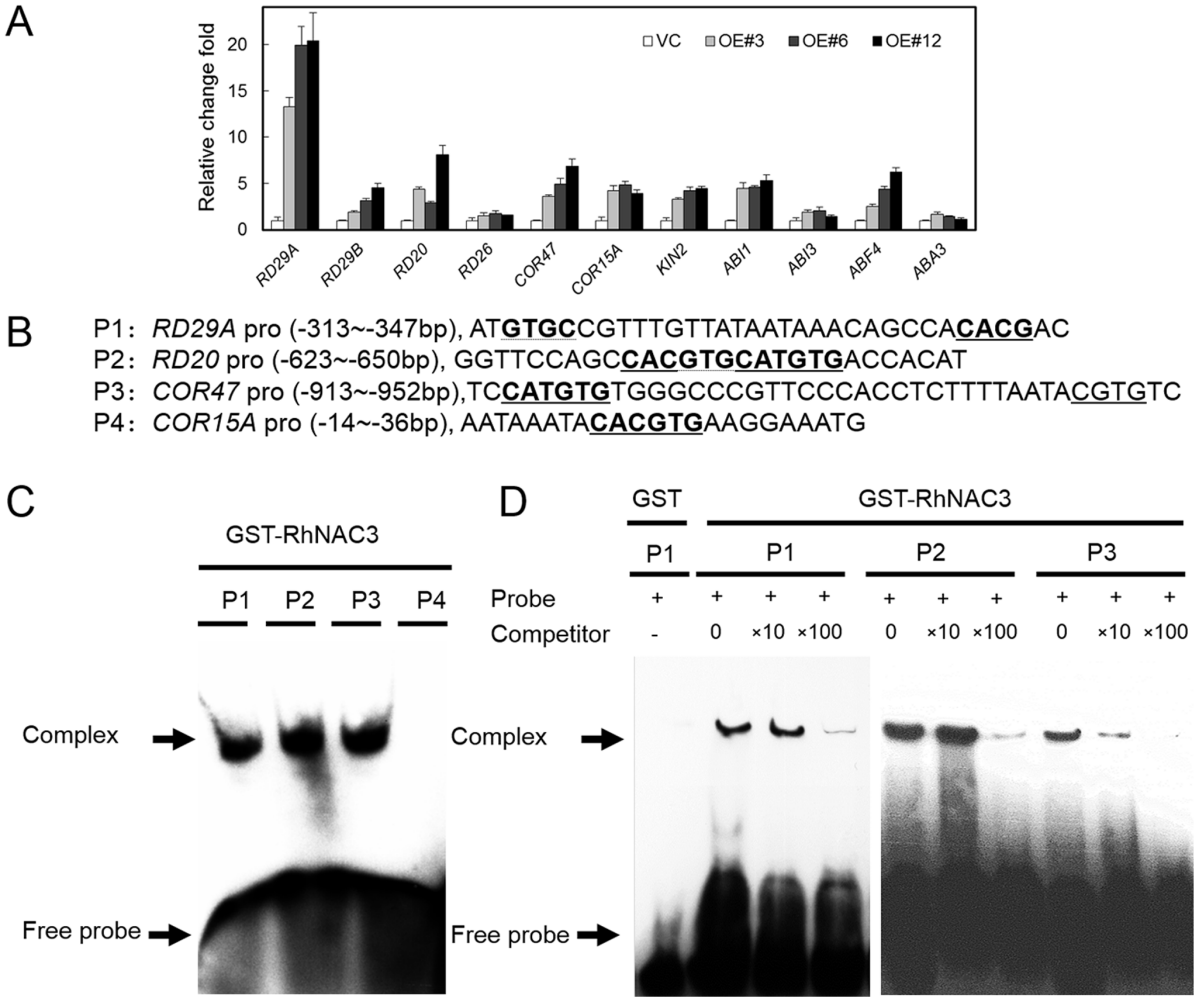
RhNAC3 binding to the regulatory sequences of ABA-related *A. thaliana* genes. **A,** ABA-related gene expression in *RhNAC3* overexpressing *A. thaliana* lines The aerial parts of light-grown, 3-week old vector control and three independent *RhNAC3* overexpressing *A. thaliana* lines (OE#3, OE#6 and OE#12) were dehydrated for 3 h and sampled (23–25°C, 40–50% relative humidity). The expression patterns of 11 ABA-responsive genes were analyzed by qPCR and the data represents the fold induction of each gene by dehydration relative to the control treatment. Mean values from three independent biological replicates were normalized to the levels of the internal control gene *Actin2*. **B,** Sequences and positions of putative RhNAC3 binding elements used in an electrophoretic mobility shift assay (EMSA). Probes were derived from the regulatory sequences of 4 selected ABA-responsive *A. thaliana* genes. Underlined letters indicate the core sequences of NAC protein targeted promoters. The sense strands of the oligonucleotide probes corresponding to the predicted RhNAC3 binding sites are shown. **C,** Interaction between GST-RhNAC3N^1–162^ and biotin-labeled probes indicated in (B). **D,** DNA-binding specificity for RhNAC3 with interacting probes. The arrows indicate the positions of protein/DNA complexes and the free probes. Purified protein (2 µg) was incubated with 0.2 pmol of the biotin probe. GST incubated with the P1 probe was used as a control, and a 10 or 100 fold excess of the unlabeled P1, P2, or P3 probes was used for competitive binding.

**Figure 7:**
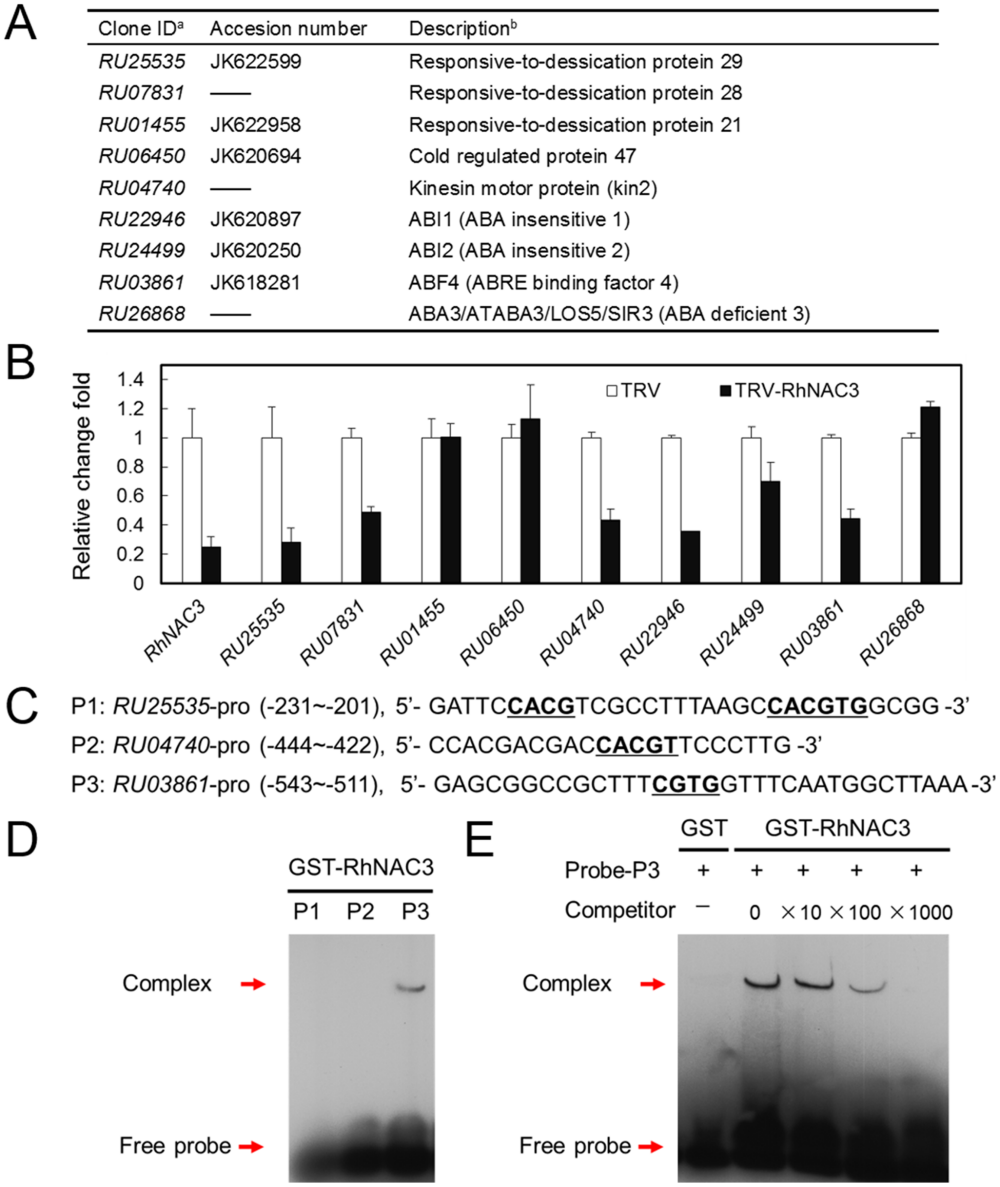
ABA-related gene expression in *RhNAC3*-silenced rose petals. **A**, The putative ABA signaling and downstream rose genes from the ABA-signaling pathway in rose. a, The clone ID from the rose transcriptome database [7]. b, Description of the *A. thaliana* homolog given by The Arabidopsis Information Resource (TAIR, http://www.arabidopsis.org). **B**, qRT-PCR analysis of *RhNAC3*-silenced rose petals. The rose cDNAs of TRV and *RhNAC3*-silenced (TRV-*RhNAC3*) petals were described in our previous report [26]. Data represent the fold change of each gene by TRV-*RhNAC3* relative to the TRV control. *RhUbi1* was used as the internal control. Error bars indicate SE (*n* = 3). **C**, Sequences and positions of putative RhNAC3 binding elements used for the EMSA. Probes were derived from the regulatory sequence of three selected ABA-related rose genes. Underlined letters indicate the core sequences of putative NAC protein-binding sites. The sense strands of oligonucleotide probes corresponding to the predicted RhNAC3 binding sites are shown. **D**, DNA-binding specificity for RhNAC3 with the probes indicated in **C**. The arrows indicate the positions of protein/DNA complexes and the free probes, respectively. Purified protein (2 µg) was incubated with 0.2 pmol of biotin probe. **E**, DNA-binding specificity for RhNAC3 with *RU03861*. The *RU03861* (P3) probe incubated with GST was used as a control, and a 10, 100, and 1000 fold excess of the unlabeled P3 was used for competitive binding.

Given that the expression of RhNAC3 showed an association with both ABA sensitivity and drought tolerance, we investigated the expression profiles of drought-induced ABA-responsive genes. Eleven representative genes were selected, including ABA and stress-induced downstream marker genes (*RD29A*, *RD29B*, *RD20*, *RD26*, *COR47*, *COR15A*, and *KIN2*), the ABA-responsive protein phosphatase 2C gene (*ABI1*), the B3-domain transcription factor ABA-insensitive 3 (*ABI3*), the ABA-activated basic Leu zipper TF gene *ABF4*, and the ABA-biosynthesis gene *ABA3* (Table 1 and Figure 6). Under dehydrating conditions, *RD26*, *ABI3* and *ABA3* showed a slightly higher level of expression in *RhNAC3* overexpressing plants than in VC plants, but all the other tested genes showed more than a two-fold greater expression (Figure 6A). The promoters of these genes were then screened for putative NAC binding *cis*-elements and indeed they were identified in all the tested genes (Table 1). We then used an electrophoretic mobility shift assay (EMSA) to investigate whether the RhNAC3 protein directly binds to 4 of the selected genes: *RD29A*, *RD20*, *COR47* and *COR15A*. We observed that RhNAC3 bound to the putative NAC recognition sites of *RD29A*, *RD20* and *COR47*, but no binding signal was detected for *COR15A*, which may therefore be an indirect target of RhNAC3 following its overexpression in *A. thaliana* (Figure 6B–D). In our previous study, we used the ATH1 microarray to identify 219 *RhNAC3*-up-regulated genes [26] and in this current study, we further analyzed the response to ABA of these genes using the AtGenExpress global stress expression dataset [35]. In total, 85 of the 219 genes were found to be ABA responsive, including those encoding proteins involved in signal transduction (e.g. calmodulin-binding protein) and TFs (e.g. zinc-finger protein) (Table 2). In rose, we found 9 putative signaling and downstream genes of the ABA-signaling pathway from our rose transcriptome databases [7] (Figure 7A). qRT-PCR analysis revealed that the expression levels of 6 of these genes were substantially repressed in *RhNAC3*-silenced rose petals (with a fold change <0.8) (Figure 7B). Three ABA-responsive genes with putative NAC binding *cis*-elements (*RU25535*, *RU04740* and *RU03861*) were selected for RhNAC3 binding assays (Table S3). EMSA revealed that RhNAC3 could bind to the promoter region of *RU03861*, a rose homolog of *ABF4*, whereas no binding signals were detected for *RU25535* and *RU04740* (Figure 7C–E), which may therefore be indirect targets of RhNAC3 in rose. Collectively, these data suggest that RhNAC3 positively activates ABA-responsive gene expression and is involved in the ABA signaling pathway in rose and *A. thaliana*.

**Table 1:** Analysis of putative NAC binding *cis*-elements in the promoter regions of genes downstream from *RhNAC3* action.

Gene name	NAC protein binding sites			
	CACG	CGTG	GTGC	CATGTG
*RD29A*	4	1	1	
*RD29B*	2	4	2	
*RD20*	7	4	2	2
*RD26*	5	6	3	
*COR47*	1	1		1
*COR15A*	4	1		
*KIN2*	6	3		
*ABI1*	2			
*ABI3*	1	2	1	
*ABF4*	3	1		
*ABA3*	4	4		

**Table 2:** Up-regulated genes involved in the ABA response in *A. thaliana* lines overexpressing *RhNAC3*.

Affy ID^a^	Description^b^	AGI Code^c^	Fold change^d^	P-value^e^
	Signal transduction (21)			
267069_at	Calmodulin (CAM)-binding protein of 25 kDa	At2g41010	6.04	0.025
258947_at	Calcium-binding EF-hand family protein	At3g01830	2.58	0.045
255844_at	Protein kinase family protein / peptidoglycan-binding LysM domain-containing protein	At2g33580	2.48	0
255503_at	Concanavalin A-like lectin protein kinase family protein	At4g02420	2.39	0.045
251054_at	Lectin receptor kinase a4.3	At5g01540	2.39	0.02
261662_at	MAP kinase kinase 7	At1g18350	2.19	0.019
266371_at	Calcium-binding EF-hand family protein	At2g41410	2.14	0.006
257751_at	MAP kinase substrate 1	At3g18690	2.09	0.011
266037_at	Protein kinase superfamily protein	At2g05940	2	0.001
	**Transcriptional regulation (32)**			
261648_at	Salt tolerance zinc finger	At1g27730	10.1	0.011
257022_at	Zinc-finger protein 2	At3g19580	7.56	0.004
248448_at	Integrase-type DNA-binding superfamily protein	At5g51190	4.41	0.04
257053_at	Ethylene responsive element binding factor 4	At3g15210	3.69	0.022
266719_at	Circadian clock associated 1	At2g46830	3.44	0.049
246932_at	Integrase-type DNA-binding superfamily protein	At5g25190	3.04	0.044
252859_at	Integrase-type DNA-binding superfamily protein	At4g39780	2.76	0.032
266656_at	Zinc finger C-x8-C-x5-C-x3-H type family protein	At2g25900	2.41	0.018
258436_at	RING/U-box superfamily protein	At3g16720	2.4	0.001
245051_at	WRKY DNA-binding protein 15	At2g23320	2.34	0.003
259626_at	Basic region/leucine zipper motif 60	At1g42990	2.26	0.007
256426_at	RING/FYVE/PHD zinc finger superfamily protein	At1g33420	2.05	0.002
252009_at	A20/AN1-like zinc finger family protein	At3g52800	2.01	0.047
	**Stress responsive (19)**			
247708_at	Zinc finger (C3HC4-type RING finger) family protein	At5g59550	4.75	0.034
257763_s_at	Receptor like protein 38	At3g23110	3.9	0.001
262911_s_at	HSP20-like chaperones superfamily protein	At1g59860	2.45	0.03
262383_at	Toll-Interleukin-Resistance (TIR) domain-containing protein	At1g72940	2.34	0.043
259105_at	Rubber elongation factor protein (REF)	At3g05500	2.22	0.044
253046_at	Cytochrome P450, family 81, subfamily D, polypeptide 8	At4g37370	2.2	0
	**Enzymes and metabolism (55)**			
254975_at	2-oxoglutarate (2OG) and Fe(II)-dependent oxygenase superfamily protein	At4g10500	7.62	0.003
256933_at	Bifunctional inhibitor/lipid-transfer protein/seed storage 2S albumin superfamily protein	At3g22600	4.07	0.005
266993_at	Major facilitator superfamily protein	At2g39210	3.58	0.005
263852_at	Nudix hydrolase homolog 6	At2g04450	3.52	0.021
252908_at	Glycolipid transfer protein (GLTP) family protein	At4g39670	3.45	0
248330_at	NAD(P)-binding Rossmann-fold superfamily protein	At5g52810	3.33	0.003
248970_at	Solute:sodium symporters; urea transmembrane transporters	At5g45380	3.12	0.003
255630_at	C2 calcium/lipid-binding plant phosphoribosyltransferase family protein	At4g00700	2.94	0.048
253332_at	Peroxidase superfamily protein	At4g33420	2.86	0.028
249188_at	HXXXD-type acyl-transferase family protein	At5g42830	2.78	0.006
266761_at	NAD(P)-binding Rossmann-fold superfamily protein	At2g47130	2.73	0.001
252098_at	Eukaryotic aspartyl protease family protein	At3g51330	2.67	0.019
253806_at	RING membrane-anchor 2	At4g28270	2.61	0.037
249910_at	Arogenate dehydratase 2	At5g22630	2.58	0.013
245035_at	Acireductone dioxygenase 3	At2g26400	2.5	0.018
251422_at	Preprotein translocase Sec, Sec61-beta subunit protein	At3g60540	2.48	0.032
267337_at	HXXXD-type acyl-transferase family protein	At2g39980	2.38	0.02
247604_at	COBRA-like protein 5 precursor	At5g60950	2.38	0.05
262237_at	Thioesterase superfamily protein	At1g48320	2.32	0
264843_at	2-oxoglutarate (2OG) and Fe(II)-dependent oxygenase superfamily protein	At1g03400	2.31	0.005
267300_at	UDP-Glycosyltransferase superfamily protein	At2g30140	2.24	0.006
253238_at	O-Glycosyl hydrolases family 17 protein	At4g34480	2.23	0.019
	**Cell expansion related (4)**			
247866_at	Xyloglucan endotransglucosylase/hydrolase 25	At5g57550	2.29	0.04
248263_at	Plant invertase/pectin methylesterase inhibitor superfamily	At5g53370	2.12	0.024
	**Others (61)**			
244966_at	Polyketide cyclase/dehydrase and lipid transport superfamily protein	At1g02470	5.58	0.041
256337_at	Serine-type endopeptidase inhibitors	At1g72060	4.84	0.008
257264_at	Receptor-like protein kinase-related family protein	At3g22060	3.77	0.015
258792_at	Glycine-rich protein	At3g04640	3.73	0.031
247193_at	MATE efflux family protein	At5g65380	3.2	0.049
250942_at	Legume lectin family protein	At5g03350	3.18	0.003
254832_at	Bifunctional inhibitor/lipid-transfer protein/seed storage 2S albumin superfamily protein	At4g12490	3.13	0.026
266097_at	SOUL heme-binding family protein	At2g37970	3.1	0.033
246289_at	VQ motif-containing protein	At3g56880	3.02	0.019
246495_at	Unknown protein	At5g16200	2.87	0.038
257690_at	SAUR-like auxin-responsive protein family	At3g12830	2.83	0.009
249769_at	Sigma factor E	At5g24120	2.75	0.027
263948_at	Late embryogenesis abundant (LEA) hydroxyproline-rich glycoprotein family	At2g35980	2.46	0.016
259502_at	Galactose oxidase/kelch repeat superfamily protein	At1g15670	2.32	0.002
248592_at	hydroxyproline-rich glycoprotein family protein	At5g49280	2.21	0.025
259410_at	Regulator of Vps4 activity in the MVB pathway protein	At1g13340	2.2	0.002
266247_at	Cysteine/Histidine-rich C1 domain family protein	At2g27660	2.15	0.019
252053_at	Syntaxin of plants 122	At3g52400	2.13	0.039
259507_at	P-loop containing nucleoside triphosphate hydrolases superfamily protein	At1g43910	2.11	0.03
264951_at	Target of Myb protein 1	At1g76970	2.11	0
262703_at	SAUR-like auxin-responsive protein family	At1g16510	2.1	0.025
258501_at	Glycine-rich protein	At3g06780	2.08	0.02
262571_at	Protein of unknown function (DUF1644)	At1g15430	2.08	0.024
251859_at	Proteophosphoglycan-related	At3g54680	2.03	0.02
	**Unknown (27)**			
253859_at	unknown protein	At4g27657	8.71	0.017
256891_at	unknown protein	At3g19030	3.98	0.031
260656_at	unknown protein	At1g19380	3.95	0.022
266017_at	unknown protein	At2g18690	3.53	0.008
265276_at	unknown protein	At2g28400	3.05	0.011
258188_at	unknown protein	At3g17800	2.57	0.021
258275_at	unknown protein	At3g15760	2.47	0.031
266259_at	unknown protein	At2g27830	2.19	0.009
252057_at	unknown protein	At3g52480	2.04	0.03

Genes derived from the *RhNAC3* up-regulated genes identified by the ATH1 microarray analysis in our previous study [26], classified to be responsive to ABA treatment according to the AtGenExpress global stress expression dataset [34].

a Affymetrix identification codes for the probes.

b Description as given by the Munich Information Center for Protein Sequences (MIPS) database.

c Represents a hyperlink to TAIR (www.arabidopsis.org) for more information.

d The ratio of three independent transgenic lines compared with the ratio of vector control plants. Genes expressed in RhNAC3 overexpressing transgenic plants with an up-regulation ratio higher than 2.0 are shown.

e Indicates one-way ANOVA of the differences in mean transcript expression levels between the transgenic and vector control plants at the 0.05 significance level.

## Discussion

### RhNAC3 is involved in the ABA-dependent signaling pathway

Plants respond and adapt to drought stresses through a broad range of molecular and biochemical processes that result in cellular physiological changes [1]. Many drought-inducible genes with various functions, including a number of TFs that regulate stress-inducible gene expression, have been identified by molecular and genomic analyses of *A. thaliana*, rice and other plants, [9], [18], [23]. These TFs have been classified as being involved in one of two signal transduction pathways: ABA-independent or ABA-dependent [2], [36]. ABA-dependent gene induction is controlled by at least five different classes of TFs at the transcriptional level: AREB (bZIPs), NACs, MYB/MYCs, AZF/STZs and DREB1D [2], [37]. Many ABA-inducible genes contain a conserved ABRE motif in their promoter regions [38], which functions as a *cis*-element in ABA-regulated gene expression. In a previous study, we determined that *RhNAC3* expression is induced by exogenous ABA [26], implying RhNAC3 is involved in the ABA signaling pathway during stress responses. In this current study, we further analyzed the *RhNAC3* promoter, which was found to contain five ABRE motifs, as well as other stress-responsive elements (Figure 1, Figure S1 and Table S2). Promoter activity was detected ubiquitously in a number of tissues upon transformation into *A. thaliana*, and was highest in leaf guard cells and some areas of the vascular system (Figure 2). In rose, *RhNAC3* expression was also detected in sepals, petals, gynoecia, stamens and receptacles [26]. ABREs were found to be important for the activity of the *RhNAC3* promoter, and the multiple copies had a cumulative effect on transcriptional activity in both the presence and absence of exogenous ABA in *A. thaliana* protoplasts (Figure 3). It has been shown that multiple ABRE elements can collectively confer ABA responsiveness to a minimal promoter, whereas a single copy of ABRE is insufficient for the full ABA response of *AREB1* and *AREB2*, two basic leucine zipper TFs [14]. ABREs are also regarded as one of the major types of *cis*-acting elements in the promoter regions of stress-inducible genes during osmotic stress-responsive transcriptional regulation [12], [39]. This is consistent with our previous findings that *RhNAC3* confers dehydration tolerance to rose petals, mainly through the regulation of osmotic adjustment-associated genes [26].

### RhNAC3 overexpression in *A. thaliana* enhances ABA sensitivity

Many ABA-responsive TFs have been isolated and characterized from different plant species, including *A. thaliana* [16], rice [5] soybean [22], maize [40] and *Citrus reshni* [41]. Overexpression of these ABA-responsive TF genes has been reported to result in a range of phenotypic changes, including dwarfing [42], [43], ABA hypersensitivity [44], lateral root formation [22] and stomatal closure [45]. In our study, *RhNAC3* overexpression in *A. thaliana* lead to ABA hypersensitivity during seedling germination and primary root growth (Figure 4), and promoted stomatal closure after exogenous ABA or drought treatments (Figure 5). We note that this differs from the effects of the soybean ABA-inducible gene *GmNAC20*, which promotes lateral root formation enhances salt and freezing tolerance when overexpressed in transgenic *Arabidopsis* [22].

### RhNAC3 enhanced ABA-responsive gene expression in rose and *A. thaliana*

ABA-inducible TFs involved in ABA signaling pathways typically up-regulate ABA-responsive genes or stress-responsive genes [13], [18] and such downstream genes have studied using qRT-PCR [16], cDNA microarrays [13] and other transcriptomic analyses [23]. Among these genes, AREB TFs play a primary role in the ABA-dependent signaling pathway [13], [46], while other TFs (NAC, MYB/MYC and AZF/STZ TFs) can play additional direct or indirect regulatory roles. The diverse *cis*-elements in the promoter regions of these TFs suggest additional potential mechanisms of transcriptional regulation for ABA-signaling downstream genes as a consequence of abiotic stresses [47]. In the current study, 11 representative ABA-induced genes were investigated, all of which were found to be up-regulated in *RhNAC3* overexpressing *A. thaliana* plants (Figure 6A). Further analysis showed that NAC binding *cis*-elements were present in the upstream regulatory sequences of these genes (Table 1) and that RhNAC3 was able to bind to the putative NAC recognition sites of some of the tested genes (Figure 6B–D). We conclude that RhNAC3 may therefore directly or indirectly regulate their expression at the transcriptional level. These genes were selected based on their invovlement in the ABA-dependent signaling pathway and the fact that their overexpression in *A. thaliana* has been shown to result in an increased ABA sensitivity [25], [48]. In a previous microarray study we found that the expression of 219 genes was up-regulated in *RhNAC3* overexpressing plants [26], of which 85 responded to ABA treatment in the current study (Table 2). These results suggest that these genes may contribute to ABA sensitivity in the *RhNAC3* overexpressing *A. thaliana* plants, an idea that is supported by previous experimental evidence. For example, *CYP81D8* expression has been suggested to be regulated by the ABA-dependent pathway under osmotic stress conditions [49], and loss-of-function mutations of *atrdufs* (At5g59550) resulted in hyposensitivity to ABA and reduced tolerance to drought stress [50]. Moreover, the expression of downstream genes in the ABA-signaling pathway was also repressed in *RhNAC3*-silenced rose petals (Figure 7). In our previous study, RhNAC3 was shown to bind to the promoter of *RU23063*, a rose homolog of *ABI2* [26], which encodes a protein phosphatase 2C involved in ABA signal transduction [51]. Here RhNAC3 was observed to bind to the promoter region of the ABA-responsive rose gene *ABF4*, which encodes the ABRE binding factor 4 (Figure 7C–E). Taken together, the data suggest that *RhNAC3* regulated genes that were responsive to osmotic stress, are also involved in the ABA-dependent signaling pathway in both rose and *A. thaliana.*

In conclusion, we found that: (1) ABRE elements in the *RhNAC3* promoter were necessary for, and had a cumulative effect on, its transcription activity in both the presence and absence of exogenous ABA; (2) *RhNAC3*-overexpressing *A. thaliana* lines showed ABA hypersensitivity during seed germination and constitutive leaf stomatal closure under ABA or drought treatment; and (3) RhNAC3 up-regulated the expression level of ABA-responsive genes, which were responsive to osmotic stress. These findings provide new evidence that RhNAC3 is a positive mediator of ABA signaling in the regulation of drought stress tolerance in rose and at least some components of the associated signaling pathways are conserved between rose and *A. thaliana*.

## Supporting Information

Figure S1 **The promoter sequence of the** ***RhNAC3*** **gene.** A cumulative result of the 1447 bp promoter sequence showing the positions of important putative cis-acting elements deduced from PlantCARE and PLACE database. The regulatory elements identified by the programs are colorful boxed with appropriate annotations. TATA: TATA box, CAAT: CAAT box, ABRE: ABRE element, CBF: cold binding factor, MYB: MYB binding site, MYC: MYC binding site, WBOX: WRKY binding site.
(TIF)

Table S1 **Primer sequences used in this study.**
(DOCX)

Table S2 ***cis*** **-elements of the upstream regulatory region of** ***RhNAC3*** **.**
(DOCX)

Table S3 **Upstream regulatory region of three ABA-related rose genes.**
(DOCX)
