# Supplementary figures and images for: The Rose (Rosa hybrida) NAC Transcription Factor 3 Gene, RhNAC3, Involved in ABA Signaling Pathway Both in Rose and Arabidopsis
Source: PLoS One. 2014 Oct 7;9(10):e109415. doi: 10.1371/journal.pone.0109415 (PMC4188598; doi:10.1371/journal.pone.0109415)

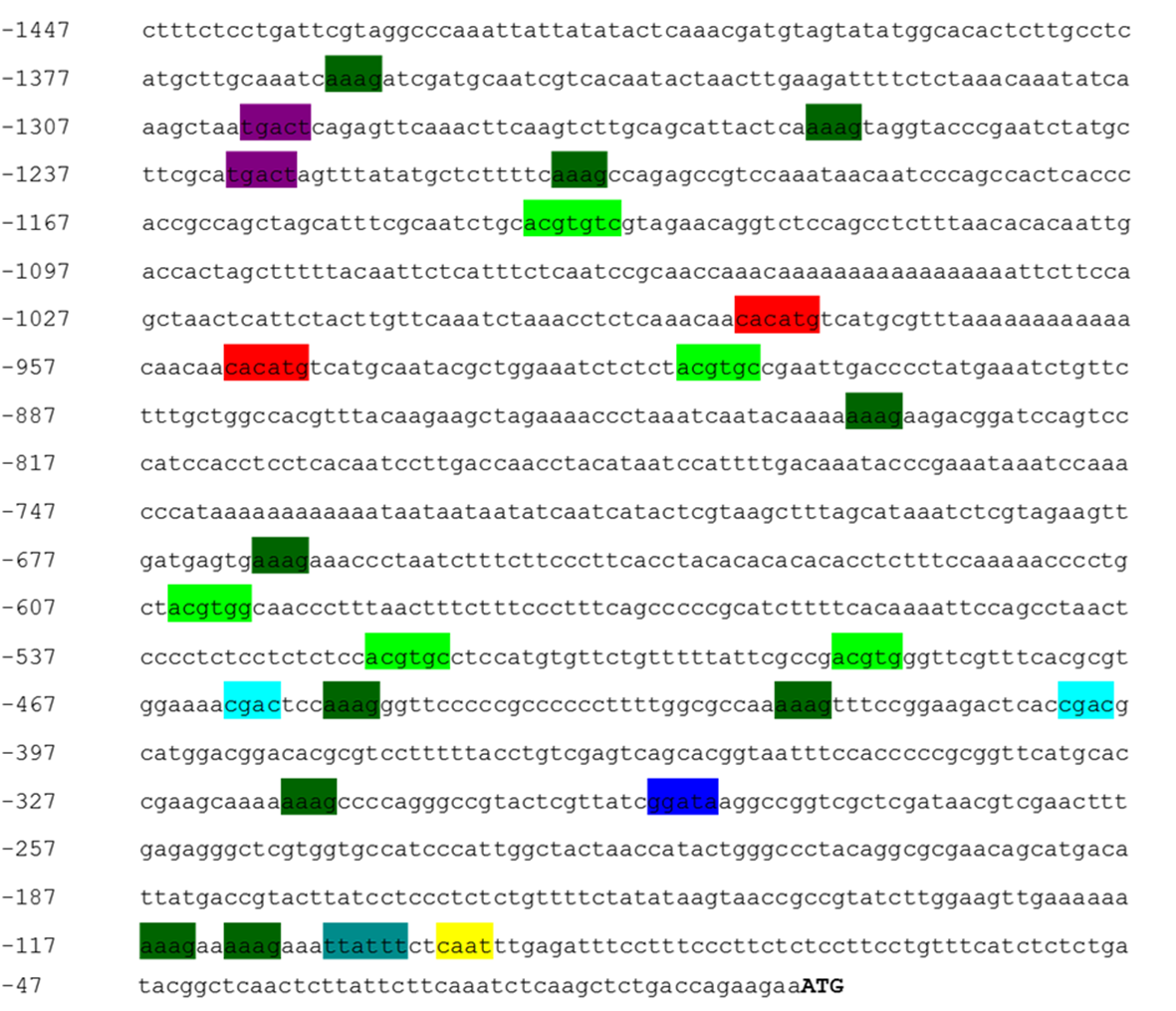

Supplement: Figure S1 — The promoter sequence of the RhNAC3 gene. A cumulative result of the 1447 bp promoter sequence showing the positions of important putative cis-acting elements deduced from PlantCARE and PLACE database. The regulatory elements identified by the programs are colorful boxed with appropriate annotations. TATA: TATA box, CAAT: CAAT box, ABRE: ABRE element, CBF: cold binding factor, MYB: MYB binding site, MYC: MYC binding site, WBOX: WRKY binding site. (TIF) [file pone.0109415.s001.tif]
