# Supplementary material for: The Rose (Rosa hybrida) NAC Transcription Factor 3 Gene, RhNAC3, Involved in ABA Signaling Pathway Both in Rose and Arabidopsis
Source: PLoS One. 2014 Oct 7;9(10):e109415. doi: 10.1371/journal.pone.0109415 (PMC4188598; doi:10.1371/journal.pone.0109415)
Supplement: Table S1 — Primer sequences used in this study. (DOCX) [file pone.0109415.s002.docx]

**Table S1.** Primer sequences used in this study.

| **Primer** | **Forward sequence（5＇-3＇）** | **Reverse sequence（5＇-3＇）** |
| --- | --- | --- |
| **For isolation of *RhNAC3* upstream regulatory sequence** | | |
| SP1 |  | GTCTGTAGGATAGAACCGAAACCCC |
| SP2 |  | TCCGGCACGCCCATTTCTT |
| SP3 |  | CAGGTTCTCTAGCCACAGAGTTGC |
| **For vector construct of *RhNAC3* promoter in GUS staining** | | |
| RhNAC3-P | ACC*AAGCTT*CATTCTACTTGTCCAAATCTGAACCTC | GC*TCTAGA*CCGTATCAGAGAGATGAAACAGGAA |
| **For qRT-PCR analysis in Arabidopsis** | | |
| *RhNAC3* | TCCGAAAGGAACCAAAACCAA | GACACAGCACCCATTCATCCA |
| *RD29A* | GCCGACGGGATTTGACG | GCCGGAAATTTATCCTCTTCTGA |
| *RD29B* | ACGAGCAAGACCCAGAAGTT | AGGAACAATCTCCTCCGATG |
| *RD20* | TTAGCTCCGGTCACCAGTCA | CATGTATGGTTTTGGTAATGTTTCC |
| *RD26* | AGTTCGATCCTTGGGATTTG | ACCCGTTGCTTTCCAATAAC |
| *COR47* | ACAAGCCTAGTGTCATCGAAAAGC | TCTTCATCGCTCGAAGAGGAAG |
| *COR15A* | AACGAGGCCACAAAGAAAGC | CAGCTTCTTTACCCAATGTATCTGC |
| *KIN2* | GCAACAGGCGGGAAAGAGTAT | CCGGTCTTGTCCTTCACGAA |
| *ABI1* | CGCTAACTGCGGTGACTCTA | CAATCCTCGCAGCTTCATCT |
| *ABI3* | ACGGGAGGGACCTGGATGTAT | CCGGCAAGTGTGTCTCAGCT |
| *ABF4* | TAGGAGGTGGTGGTCATCCT | CCGGTCCACCTAGTGTGTT |
| *ABA3* | GGATCATGCTGGTTCTACTTTG | TGTCGAGCATCCGCTATAAG |
| *ACTIN2* | CTAAGCTCTCAAGATCAAAGGC | AACATTGCAAAGAGTTTCAAGG |
| **For qRT-PCR analysis in *RhNAC3*-silenced rose petals** | | |
| *RU25535* | CAGGCACAGAGTCCCTCATC | GCGACTAGGAGGACTGATGC |
| *RU07831* | AGTGTAGCATCCGCCTGTCT | TCCACAAGTCCGAGGAACCA |
| *RU01455* | ATCTGGGTAACACCGCAACC | TTGAAGGTTTCTCGGGGCAG |
| *RU04740* | TGCGGAGTGAGTATCCTGAGA | TGATCTGGTTTCCATGTCCCA |
| *RU22946* | TCCTACGCTTTCGATCATGTGT | ATCTGTTTCGGAGCCATTCA |
| *RU24499* | AGCACTTTGGACCGTGGTAG | GTCCCTTCAGCCCTTTGACT |
| *RU03861* | TTGTCGTGGCAAACAACCAG | ATTGCATGACGGGGTAGAGT |
| *RU26868* | GCTTCTACCTCGGACCCTTT | TCTCTCTTTTGGGTCTGAAGC |
| *RU25535* | GACTGTATAGCTGTGGTGCC | TCGAAACCCAGTAGGGGTGA |
| *RhUBI1* | GGGCAATCATCTGGAAGTGCTCGT | GCCCCCAAAGAGAAACCCTGCG |
| **For vector constructs of GUS activity in Arabidopsis protoplasts** | | |
| N0 | ACC*AAGCTT*CTTTCTCCTGATTCGTAGGCC | TA*CTGCAG*CAGAGAGGGAGGATAAGTACGG |
| N1 | ACC*AAGCTT*CGTAAGCTTTAGCATAAATCTCGT |  |
| N2 | ACC*AAGCTT*GGCCGTACTCGTTATCGGATAAGG |  |
| mN0-A1 | GTCgtagaacaggtctccagcctc | CCTGTTCTACGACtaaaGCAGATT |
| mN0-A2 | GCCGAATTGACCCCTATGAAAT | GGTCAATTCGGCtaaaAGAGAGA |
| mN0-A3 | GGCAACCCTTTAACTTTCTTTCC | GGTTGCCtttaAGCAGGGGTTTTTG |
| mN0-A4 | ATTTGCCTCCATGTGTTCTGT | GGAGGCtttaGGAGAGAGGAGA |
| mN0-A5 | TAGGGTTCGTTTCACGCGT | CGAACCCtttaCGGCGAATA |
| **For vector construct of RhNAC3 in EMSA** | | |
| *RhNAC3-*EMSA | CCA*GGATCC*ATGGGCGTGCCGGAAAC | TA*GAGCTC*GCTTCTTGTAAATACGACACAGCACC |
