# Supplementary material for: The Rose (Rosa hybrida) NAC Transcription Factor 3 Gene, RhNAC3, Involved in ABA Signaling Pathway Both in Rose and Arabidopsis
Source: PLoS One. 2014 Oct 7;9(10):e109415. doi: 10.1371/journal.pone.0109415 (PMC4188598; doi:10.1371/journal.pone.0109415)
Supplement: Table S2 — cis -elements of the upstream regulatory region of RhNAC3 . (DOCX) [file pone.0109415.s003.docx]

**Table S2.** *cis*-elements of the upstream regulatory region of *RhNAC3*.

| Motif | Consensus | Location | Description | Reference |
| --- | --- | --- | --- | --- |
| CAAT-box | CAAT | -106/-104 | Tissue specific | [1] |
| TATA-box | TTATTT | -104/-99 | Critical for accurate initiation | [2] |
| ABRE | ACGTG | -1140/-1136, -918/-914, -605/-601, -521/-517, -488/-484 | ABA responsive element; Responsive to dehydration | [3-4] |
| CBF | RYCGAC | -493/-488, -404/-399 | Dehydration-responsive element binding proteins | [5-6] |
| Myb-type | GGATA | -291/-287 | Water stress; Regulation of flavonoid biosynthesis | [7] |
| Myc-type | CACATG | -985/-980, -951/-946 | Dehydration-responsive; ABA-induction | [8-9] |
| W-box | TGACT | -1300/-1296, -1231/-1227 | Sugar-responsive | [10] |

The major *cis*-elements sequences and positions of *RhNAC3* promoter region are shown and corresponding to those presented in Figure S1.

**References**

1. Shirsat A, Wilford N, Croy R, Boulter D (1989) Sequences responsible for the tissue specific promoter activity of a pea legumin gene in tobacco. Molecular and General Genetics MGG 215: 326–331.
2. Tjaden G, Edwards JW, Coruzzi GM (1995) Cis elements and trans-acting factors affecting regulation of a nonphotosynthetic light-regulated gene for chloroplast glutamine synthetase. Plant Physiol 108:1109–1117.
3. Nakashima K, Fujita Y, Katsura K, Maruyama K, Narusaka Y, et al. (2006) Transcriptional regulation of ABI3-and ABA-responsive genes including *RD29B* and *RD29A* in seeds, germinating embryos, and seedlings of *Arabidopsis*. Plant Mol Biol 60: 51–68.
4. Simpson SD, Nakashima K, Narusaka Y, Seki M, Shinozaki K, et al. (2003) Two different novel *cis*-acting elements of *erd1*, a clpA homologous Arabidopsis gene function in induction by dehydration stress and dark-induced senescence. Plant J 33: 259–270.
5. Svensson JT, Crosatti C, Campoli C, Bassi R, Stanca AM, et al. (2006) Transcriptome analysis of cold acclimation in barley Albina and Xantha mutants. Plant Physiol 141: 257–270.
6. Xue GP (2002) Characterization of the DNA‐binding profile of barley HvCBF1 using an enzymatic method for rapid, quantitative and high-throughput analysis of the DNA-binding activity. Nucleic Acids Research 30: e77–e77.
7. Baranowskij N, Frohberg C, Prat S, Willmitzer L (1994). A novel DNA binding protein with homology to Myb oncoproteins containing only one repeat can function as a transcriptional activator. The EMBO journal 13: 5383.
8. Abe H, Yamaguchi-Shinozaki K, Urao T, Iwasaki T, Hosokawa D, Shinozaki K (1997) Role of Arabidopsis MYC and MYB homologs in drought-and abscisic acid-regulated gene expression. Plant Cell 9: 1859–1868.
9. Busk PK, Pages M (1998) Regulation of abscisic acid-induced transcription. Plant Mol Biol 37: 425–435.
10. Sun C, Palmqvist S, Olsson H, Borén M, Ahlandsberg S, et al. (2003) A novel WRKY transcription factor, SUSIBA2, participates in sugar signaling in barley by binding to the sugar-responsive elements of the *iso1* promoter. Plant Cell 15: 2076–2092.
