# Supplementary material for: The Rose (Rosa hybrida) NAC Transcription Factor 3 Gene, RhNAC3, Involved in ABA Signaling Pathway Both in Rose and Arabidopsis
Source: PLoS One. 2014 Oct 7;9(10):e109415. doi: 10.1371/journal.pone.0109415 (PMC4188598; doi:10.1371/journal.pone.0109415)
Supplement: Table S3 — Upstream regulatory region of three ABA-related rose genes. (DOCX) [file pone.0109415.s004.docx]

**Table S3** Upstream regulatory region of three ABA-related rose genes.

The putative core DNA sequence of CGT[G/A] or its reversed complementary [T/C]ACG of NAC binding are shown in red fonts with underlined. The fragments in red box show the probe used in EMSA assay in Figure 7.

1. The upstream regulatory region of *RU25535* (*Responsive-to-dessication protein 29*)

-480 acgatggactccagagcggccgcccggatggtttctctgactagctactggacggctgctgctaacagaaagtaatgtcactgcaattat

-390 tttcatttctgcagcaatcccatcatatacagtaccacgtcttcatgtccgacctatccacaactccatctcaccccgaatacgacgtcg

-300 cttaagacctgggcaatcagtaacggcaagtcggcaacttactgtgtgattatccattgcaggagaaacgattc***cacg***tcgcctttaagc

P1

-210 ca***cgtg***gcggaaaatgagacttggttggtttatttcgttactcgtgtcgttcatatagagcccgacacatgcagaggaggcacaaccttt

-120 tgctcccgccatccctttaaagcttccccttcccctcctctgtttctcacactcttgtttctttcaacacaaaaaaaattctttgtttta

-30 atcaatcagaaatatcgcaaagtttggatcATGGATACTCATGCAGTACATTCTCGTGTT

1. The upstream regulatory region of *RU04740* (*Kinesin motor protein* (*kin2*))

-778 ggccggatcaataatgcagctggcatgacaggtttcccgagtgaaagcgggcagtgagcgcaacgcaattaatatgagtgagc

-695 tcactcattaggcaccccaggctttacactttatgcttccaggctagtatgttgtgtggaaattgtgagcgggttaacgattt

-612 cacacagggaaacagctatgaccatgattacgccaagctatttaggtgactctatagaatactcaagctatgcatccaacgcg

-529 ttgggagctctcccatatggtcgacctgcaggcggccgcgaattcactagtgattacgaggactccagagcggccgcacagtg

-446 cac***cacg***acgac***cacg***ttcccttgcttccgtatatgttcaataaactcttcctctcttcatgtgctagctgacacactcgcca

P2

-363 attgccctaatgacaagttgaaccaccccttccccccacccctactataattatcatctccctcctctccctcctttcatttc

-280 ttcctccgtctccgtccttcttattatatactaggttcctatatatggttaacctctcttccttctcaccatcaccatgtcct

-197 ccaacttctccctcccaaggtttcatgtcctctactcatttatcgtatctattttaattaaaattcgagggcgggctttccga

-114 agtgctggtctcttcttctgtagacgcttccggtgaattgaccagcaccgagagctaagcaaagctaactgaatcctcttaat

-31 taaattttgaaactaacaatattttacgtgcATGATTATATTTGCTCTAACTGTGTTACACAT

1. The upstream regulatory region of *RU03861*(*ABF4* (*ABRE binding factor 4*))

-628 ggtaactctatagggcgattgggcccgacgtcgcatgctcccggccgccatggcggccgcgggaattcgattacgatggactcca

-543 gagcggccgcttt***cgtg***gtttcaatggcttaaaagccaaagggggactggcgaaagagctgcagacacttggcgctagcagcaca

P3

-458 gccctaaacgcaagcaccaattttcagcagccacacagacgccaaagttcaaaaattcccaaaaatagtaaccaggcaaaaagaa

-373 agcctatattaggaatttttattaatttgtttggataatttatttcttcttcatcctgagcctgagggtttatgtgtgtgttaat

-288 ctgtatttgctggaaattttgagctgggaaacttggtaggttttggttttggttttgttgtttgctcttgttcttgtttttgcat

-203 gagaaaactgagcttttggcaagtgatttggagtaaatcactgattagggtttaatgagctgaagcgtgtgtgtgtgaacatttt

-118 gattggctgaaacttgaaaatggtcaatttctgtgagcttaaatgagaatatatcaaagtatttcccaattttccaggtttgaat

-33 tagaagctttagccctttgtttcagtgacacacATGGGTGCCAATATGAA
